# Application of Omics Tools in Designing and Monitoring Marine Protected Areas For a Sustainable Blue Economy

**DOI:** 10.3389/fgene.2022.886494

**Published:** 2022-06-22

**Authors:** Nicholas W. Jeffery, Sarah J. Lehnert, Tony Kess, Kara K. S. Layton, Brendan F. Wringe, Ryan R.E. Stanley

**Affiliations:** ^1^ Bedford Institute of Oceanography, Fisheries and Oceans Canada, Dartmouth, NS, Canada; ^2^ Northwest Atlantic Fisheries Centre, Fisheries and Oceans Canada, St. John’s, NL, Canada; ^3^ School of Biological Sciences, University of Aberdeen, Aberdeen, United Kingdom

**Keywords:** marine conservation, population genomics, environmental DNA (eDNA), metabarcoding, connectivity, conservation planning

## Abstract

A key component of the global blue economy strategy is the sustainable extraction of marine resources and conservation of marine environments through networks of marine protected areas (MPAs). Connectivity and representativity are essential factors that underlie successful implementation of MPA networks, which can safeguard biological diversity and ecosystem function, and ultimately support the blue economy strategy by balancing ocean use with conservation. New “big data” omics approaches, including genomics and transcriptomics, are becoming essential tools for the development and maintenance of MPA networks. Current molecular omics techniques, including population-scale genome sequencing, have direct applications for assessing population connectivity and for evaluating how genetic variation is represented within and among MPAs. Effective baseline characterization and long-term, scalable, and comprehensive monitoring are essential for successful MPA management, and omics approaches hold great promise to characterize the full range of marine life, spanning the microbiome to megafauna across a range of environmental conditions (shallow sea to the deep ocean). Omics tools, such as eDNA metabarcoding can provide a cost-effective basis for biodiversity monitoring in large and remote conservation areas. Here we provide an overview of current omics applications for conservation planning and monitoring, with a focus on metabarcoding, metagenomics, and population genomics. Emerging approaches, including whole-genome sequencing, characterization of genomic architecture, epigenomics, and genomic vulnerability to climate change are also reviewed. We demonstrate that the operationalization of omics tools can enhance the design, monitoring, and management of MPAs and thus will play an important role in a modern and comprehensive blue economy strategy.

## Introduction

**FIGURE 1:**
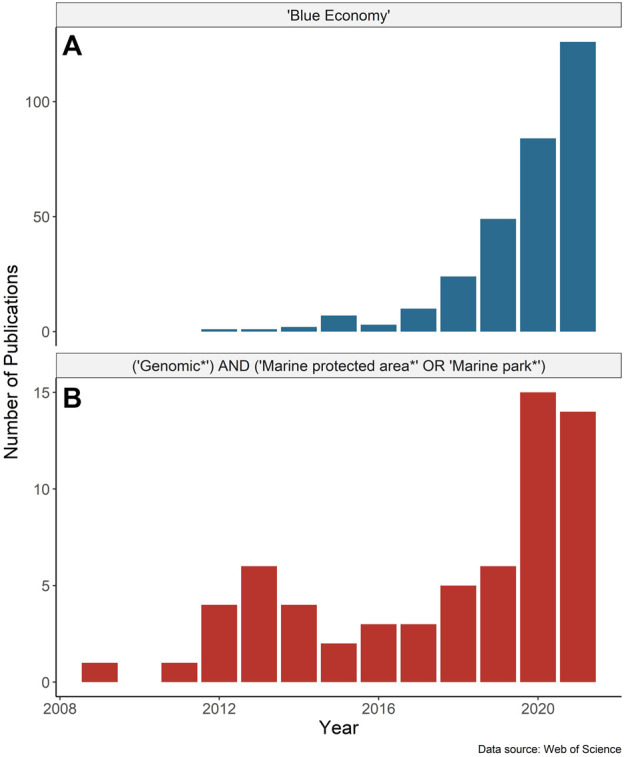
Number of publications by year based on the Web of Science search for papers with any field containing **(A)** “Blue Economy” and **(B)** “Genomic*” AND (“Marine protected area*” OR “Marine park*”). Search results were accessed on February 18, 2022, and include only publications up to the end of 2021.

The global “blue economy” strategy (BES) centers on the long-term sustainable use of ocean resources to promote economic benefits while also preserving ocean ecosystems (Smith-Godfrey 2016). This emerging approach integrates the conservation of ecosystem services, including economic activities, tourism, transportation, fishing, and resource extraction, with marine spatial planning and conservation to sustain the health of wild populations and marine ecosystems (Bennett et al., 2019). The application of the BES has been gaining momentum as has the supporting research (Figure 1). Marine protected areas (MPAs) are centered in the global effort to safeguard biological diversity and thus are integral to the BES (Gaines et al., 2010; Agardy et al., 2011). The pace of MPA establishment is increasing globally, catalyzed by international agreements and conservation targets (e.g., Aichi Target 11, the “*30by30*” target initiative; Global Ocean Alliance 2022). As of 2022, nearly 8% of the global ocean falls under some form of spatial-based marine biodiversity conservation measure (UNEP-WCMC and IUCN 2022) with the expansion expected to continue.

Marine protected areas represent a spatial tool for the conservation of biodiversity, and fit into a broader approach for ecosystem-based management and spatial planning (Halpern et al., 2010). MPAs can contribute to the blue economy by protecting unique or vulnerable populations (e.g., Morris and Green, 2014) from anthropogenic impacts such as overharvesting (Morris et al., 2014; Sinclair-Waters et al., 2018b), by supplementing fisheries management (e.g., Gaines et al., 2010), and/or by enhancing “blue” carbon sequestration (Macreadie et al., 2021). When established as a functioning, connected network, MPAs can help to conserve important ecosystem functions and enhance population productivity beyond their legislated boundaries (Harmelin-Vivien et al., 2008; Grorud-Colvert et al., 2014; Di Lorenzo et al., 2020). Healthy marine ecosystems can also provide the indirect benefit of helping to mitigate impacts of climate change by protecting against other stressors and providing habitat refugia (e.g., Tittensor et al., 2019; Bryndum-Buchholz et al., 2022). The rapid global expansion in the global coverage of MPAs, which increasingly are being applied at a large spatial scale and in remote locations (e.g., Wilhelm et al., 2014), necessitates new, scalable technologies. Emerging omics approaches are uniquely suited to address this problem and help inform the design, monitoring, and successful implementation of MPAs; however, the incorporation of these approaches into MPA research is still in its infancy (Figure 1). Omics approaches are generally described as high-throughput technologies to holistically sequence or quantify DNA, RNA, proteins, metabolites, and other molecules, and include (meta)genomics, (meta)transcriptomics, epigenomics, lipidomics, metabolomics, and proteomics, among others (*sensu* Samuel et al., 2021).

Here, we provide an overview of molecular omics approaches, focusing on genomics, transcriptomics, and environmental DNA (eDNA) metabarcoding, and emphasize how development and application of such methods can address key needs for the successful design and management of MPAs to support the goals of a blue economy. We demonstrate how omics tools can be used to characterize genetic diversity, evaluate functional connectivity, identify adaptive variation and environmental association, and to predict population-level responses to climate change. These tools can reveal fine-scale evolutionary processes required for conservation planning that were previously either not considered or not detectable (Xuereb et al., 2019). We also explore how metagenomics and eDNA metabarcoding are powerful approaches for baseline characterization of eukaryotic and microbial diversity, helping to inform ecological status and the long-term management of protected areas. We focus our perspective through the application of conservation areas within a BES. While other omics approaches (e.g., metabolomics) have applications for BES, they were outside the scope of this review.

### Applications of Omics for Marine Protected Area Planning

Representativity and connectivity are core design principles when establishing MPA networks to ensure that the protection is equitably distributed through space. Genomic approaches, such as reduced representation genome sequencing (e.g., restriction site–associated DNA sequencing, RAD-seq; Davey and Blaxter, 2010), pooled sequencing (PoolSeq; Schlötterer et al., 2014), and low-coverage whole-genome resequencing (lcWGS; Lou et al., 2021), have considerably advanced our understanding of genetic diversity and population connectivity in non-model species over the last decade. Characterizing genome-wide variation through genomic sequencing has revealed cryptic intraspecific population structure where panmixia has otherwise been assumed, informing both representativity and connectivity for conservation network design (e.g., Barney et al., 2017; Van Wyngaarden et al., 2017).

MPA networks that consider connectivity and gene flow in their design will be better positioned to conserve diversity and adaptive variation within species (von der Heyden 2009), while also protecting genetically distinct populations (Andrello et al., 2022). Explicit consideration of genetically unique or diverse populations can help maintain diversity, and may allow for potential genetic rescue or the re-establishment of populations following extirpation (Xuereb et al., 2019), thus increasing resilience *via* the conservation of biocomplexity (Mendez et al., 2014). Movement of individuals between protected areas can occur through active migration or passive dispersal (e.g., Roberts et al., 2021), in some cases resulting in gene flow, as well as contributing to productivity and recruitment to adjacent fished stocks (e.g., Huserbråten et al., 2013). Genomic tools can provide empirical evidence of connectivity, which is difficult to quantify with conventional monitoring approaches for many taxa (Balbar et al., 2020). For example, genetic studies assessing kinship in Australasian snapper (*Chrysophrys auratus*) revealed that reproductive productivity within an MPA disproportionately contributed to recruitment in surrounding areas (Le Port et al., 2017). In other cases, genetic methods have revealed evidence of selection for limited dispersal, which correspondingly drove fine-scale genetic structure associated with the MPA protective measures (Sanford et al., 2006; Baskett and Barnett, 2015).

Often, fine-scale population differentiation identified in marine species is driven by adaptation to gradients in physical and chemical ocean conditions (e.g., Stanley et al., 2018). Recent genomic studies have revealed molecular underpinnings of local adaptation to the environment (e.g., temperature, salinity, and oceanography). Adaptive traits may be underlain by genetic architectures ranging from many small-effect loci (Bay R. A. et al., 2017) to single large-effect genes (Barson et al., 2015; Prince et al., 2017). The genetic architecture underlying local adaptation is predicted to affect how species respond to both current conditions and future change, making quantification of adaptive variation essential information for building resiliency into conservation planning (Bay R. A. et al., 2017; Lowen et al., 2019; Oomen et al., 2020; Layton and Bradbury 2022). Genomic data can also reveal other types of variation in the genome that play key roles in adaptation and population structure and persistence; this variation could inform conservation design and be used for monitoring. For example, Catanach et al. (2019) demonstrated that structural variants in the Australasian snapper genome outnumbered the SNP associated variation based on total bases affected. Structural variation such as large chromosomal inversions (Kess et al., 2019) and copy number variants (CNVs) (Kess et al., 2021; Layton et al., 2021) have also been identified in aquatic systems underlying divergent ecotypes and genomic signals of climate adaptation. By identifying and incorporating information on adaptive variation into conservation planning, specific areas can be prioritized to help protect biodiversity and promote long-term persistence of populations under climate change (Xuereb et al., 2021).

Recent genomic studies have also revealed the magnitudes of population decline using measures of genetic diversity such as effective population size (*N*_e_), and have uncovered genome-wide differences between declining and stable populations (Hollenbeck et al., 2016; Lehnert et al., 2019). These types of analyses hold promise to identify species and populations of conservation concern for MPA design. Similarly, metrics of genetic diversity and connectivity, including *N*_e_ and heterozygosity, can be incorporated into conservation planning through network objectives (e.g., Gajdzik et al., 2021) or planning tools such as *Marxan*. In coastal Africa, Phair et al. (2021) demonstrated that conservation planning based on habitat models alone risked missing important genetic variation in the coastal seagrass (*Zostera capensis*) leading to design configurations that did not include evolutionarily unique populations and were thus less resilient to environmental change. These results emphasize the value of collecting genetic information *a priori*. Incorporating population-level stratification into the MPA network design can promote the conservation of genetic diversity and resiliency following the stewardship aspects of the BES.

While the theoretical applicability of omics tools in the MPA design and monitoring are robust, few real-world examples exist. One example of genomic application to MPA research focuses on the Gilbert Bay MPA in Labrador, Canada. With the collapse and subsequent moratorium of the Atlantic cod (*Gadus morhua*) came an increased pressure on nearshore cod stocks. A phenotypically and genetically distinct population of inshore cod reside within the Gilbert Bay MPA, which was established to protect this population (DFO 2010). Though the MPA protected the core habitat, genomic stock identification revealed that these genetically divergent cod were being exploited in adjacent fisheries (Sinclair-Waters et al., 2018a,b). In this case, genomic data corroborated prior tagging data (Morris et al., 2014) but also revealed the adaptive mechanism (i.e., chromosomal inversions) of divergence between the Gilbert Bay and northern cod (Sinclair-Waters et al., 2018a). In the long-term, the ability to genetically assign catch in fisheries outside the MPA will provide an important and robust basis for MPA management. Another study conducted on the California sea cucumber (*Parastichopus californicus*) used genomic data to identify priority areas for MPAs (Xuereb et al., 2021). The study found that focal areas for conservation differed among genomic metrics (i.e., diversity vs adaptive variation), highlighting the need to match the genetic metrics with the conservation objectives when evaluating design decisions. In each example, the application of genomic approaches revealed novel information about the conservation priorities that were directly applicable to design and monitoring.

### Applications of Omics for Marine Protected Area Monitoring

Spatial conservation tools like MPAs help to regulate anthropogenic impacts on populations, and these impacts can be monitored using molecular omics approaches. Short-term human impacts, including overfishing, aquaculture escape events, and pollution, have produced measurable differences in adaptive variation among populations (Wringe et al., 2018; Therkildsen et al., 2019; Phair et al., 2020), demonstrating that omics have utility in quantifying anthropogenic impacts within and outside of MPAs. Transcriptomic methods (i.e., RNA-sequencing) can identify differences in gene expression among individuals, including variation in the transcript level and splicing, and can be used to identify mechanisms of rapid adaptation (Jacobs and Elmer, 2021). For example, Baratti et al. (2022) demonstrated higher concentrations of contaminants in marbled crab (*Pachygrapsus marmoratus*) from a polluted port compared to those in an adjacent MPA, and RNA-sequencing supported corresponding differences in the expression of stress-related genes supporting the conservation efficacy of the MPA. Epigenetic changes, such as DNA methylation, can provide another heritable means by which organisms may alter their phenotype and rapidly respond to environmental or anthropogenic stressors without changes to their underlying DNA sequence (Anastasiadi et al., 2021). As technologies evolve to accurately characterize genomic variation associated with phenotype, greater capacity to quantify and mitigate impacts of stressors, predict population responses, and ultimately conserve genetic biodiversity will improve.

A growing area of omics application and research is eDNA metabarcoding, which has revolutionized biomonitoring in aquatic ecosystems through detection of invasive and/or rare species (Weltz et al., 2017; Matejusova et al., 2021) and the characterization of community-level biodiversity (Bani et al., 2020; Djurhuus et al., 2020). This method involves the collection of extracellular DNA from aquatic samples and is often more taxonomically and monetarily efficient than traditional monitoring approaches (Fediajevaite et al., 2021; He et al., 2022). eDNA collection is minimally invasive and can be implemented in various environments and conditions, and thus represents a useful tool for MPA monitoring (Sawaya et al., 2019; Gold et al., 2021). Globally, MPAs cover millions of square kilometers, and scalable, cost-effective tools such as metabarcoding represent a tractable efficient solution for monitoring at large spatial extents and in remote areas. Traditional survey-based methods (e.g., trawling) are time-intensive and often limited by depth or topography, whereas eDNA sampling, while not without its own biases, is relatively simple, cost-effective, can be scaled across large MPAs (Gold et al., 2021), and provides a standardized biomonitoring platform deployable across ecosystem types (e.g., coastal to remote deep ocean).

New eDNA studies are developing PCR-free methods that employ direct sequencing of genomic DNA through shotgun metagenomic sequencing or “genome skimming” (Cowart et al., 2018), which can provide a more accurate measure of species abundance and prevent amplification biases (Peel et al., 2019). In addition, it is now common practice to sequence DNA directly in the field, using long-read Nanopore sequencing which can generate full-length gene or genome sequences to improve taxonomic resolution (Baloğlu et al., 2021). For reliable identifications, comprehensive and well-curated reference databases are critical; while such databases are publicly available and growing, many remain incomplete and can be difficult to curate (Weigand et al., 2019). In light of this, new bioinformatic approaches make use of reference-free identification algorithms, employing density-based clustering to detect both known and unknown species (Baloğlu et al., 2021). Exploration of the use of automated eDNA collection and processing systems, either mounted statically in key areas, or onboard automated vehicles is underway, providing a platform for real-time, continuous monitoring (e.g., Hansen et al., 2020). The use of marine invertebrates as natural eDNA samplers has also gained recent attention (e.g., sponges, Mariani et al., 2019). Though more work remains to test the applicability of eDNA as a surrogate for long-term monitoring approaches (Antich et al., 2021; Gold et al., 2021; He et al., 2022), it shows promise to be an adaptable technology for biomonitoring, which can inform management, detect species distribution shifts under climate change, and be a core part of the larger effort to sustainably manage marine activities under a BES (Curry and Ausubel 2021).

### Recommendations for Operationalization and Future Directions

Interest in the blue economy and research involving genomics in MPAs has expanded markedly since 2018 (Figure 1). The proliferation of marine omics studies and the move to open-access data repositories have the potential to contribute to the planning and monitoring of MPAs and be a key tool in fisheries management for the blue economy. Advances in genome assembly approaches (e.g., chromosome level assemblies using chromatin capture, optical mapping, and long-read sequencing) add to a growing number of high quality reference genomes, through larger initiatives such as the Vertebrate Genomes Project. As the number of resources for species grows, so does the ability to integrate datasets into larger-scale single-species studies (Jeffery et al., 2018), or multi-species research (Stanley et al., 2018; Gajdzik et al., 2021). We recommend the regular incorporation of genomic and other omics data for species of interest (i.e., at the very least, those that are conservation priorities for the network) in the design and ongoing monitoring of marine conservation areas, to understand population structure, connectivity, and species diversity (see Table 1 for a list of applications and recommendations).

**TABLE 1:** Examples of molecular omics tools and their applications in various stages of MPA design and management, including baseline data acquisition, network design to incorporate genetic diversity and connectivity, and monitoring MPAs. Recommendations for which methods or tools to use are provided, with example references. We note that this does not represent an exhaustive list of methods and applications, but can be used to guide the process of MPA design and implementation.

Recommendation	Omics approach	Data type	Examples of application (with references)
**Initial MPA design and planning**			
Measure connectivity, gene flow, and population structure to identify unique populations and specific areas or populations for conservation	Quantify genetic divergence among populations (e.g., *F*_ST_), connectivity, and effective gene flow	(Multi-species) Genomic data* sampled from multiple geographic regions or populations	Characterization of population structure and estimates of dispersal and connectivity in sea scallop using RAD-sequencing (Van Wyngaarden et al., 2017)
			Identification of high connectivity among Australian and New Zealand School sharks based on genome-wide neutral SNPs (Devloo-Delva et al., 2019)
Identify barriers to gene flow or connectivity corridors to incorporate into network design.	Clustering and characterizing population structure and admixture (e.g., STRUCTURE, ADMIXTURE, PCA, and DAPC)	Genomic data and metadata, including geography and environmental data can help explain identified population structure	Identification of a reproductively isolated cod population within an MPA based on neutral genomic divergence, using *F*_ST_ and clustering methods (DAPC and STRUCTURE) on data from an SNP array (Sinclair-Waters et al., 2018b)
			Identification of cryptic diversity and admixture in neon goby in a Belizean marine reserve network using double-digest RAD-seq (ddRAD) (D’Aloia et al., 2017)
Quantify genetic diversity within areas/populations	Estimate effective population size, (*N*_e_) using linkage disequilibrium, and/or coalescent modeling	Genome-wide data* sampled from populations and areas of interest	Estimates of ancient and contemporary *N*_e_ using SNPs of the Grey reef shark show population size increases that coincide with range expansions in the Coral Triangle (Walsh et al., 2022)
	Calculate heterozygosity, inbreeding coefficients, and allelic richness		Genome-wide sequenced microsatellites reveal self-seeding and low dispersal among corals in marine reserves in Palau (Cros et al., 2017)
Identify adaptive genetic variation within and among populations	Genome-wide association studies (GWAS)	Genomic data* in combination with phenotype data (e.g., body size, migratory ability, and color morphs), or environmental data (e.g., sea surface temperature and salinity)	Identification of loci underpinning traits of conservation interest, such as migration ecotypes in cod (Sinclair-Waters et al., 2018b; Kess et al., 2019) and age-at-maturity in salmon (Barson et al., 2015)
	Genome-environment associations (GEA)	RNA-sequencing data from populations of interest (e.g., in stressed and pristine environments)	Identification of loci associated with environment, including loci associated with temperature adaptation using genomic data (Jeffery et al., 2018; Stanley et al., 2018) or transcriptomic data (Bay and Palumbi 2014)
	Transcriptomics and gene-expression (in conjunction with other omics such as proteomics/metabolomics)		Characterization of structural variation (such as copy number variation and chromosomal rearrangement) revealed underpinnings of local adaptation to temperature in lobster (Dorant et al., 2019)
Collect baseline data on species richness and biodiversity for fish, invertebrates, macrophytes, microbes, and other taxa of interest	Metabarcoding of environmental DNA (eDNA) collected from sediment or water samples across a region of interest for cataloguing baseline diversity. Benthic and surface water samples with ≥3 replicates are recommended to capture greater diversity	eDNA sequences (long and/or short reads)	West et al. (2020) developed a multi-marker baseline catalog of marine fishes and invertebrates for a coral reef atoll to aid in ongoing monitoring and management
		Voucher specimens of species for inclusion in reference databases	
		Environmental covariates, such as water temperature, pH, and salinity	
**MPA conservation objective monitoring and management**			
Identify fishing pressure on species within and outside MPA boundaries	Genetic stock identification (GSI) to quantify dispersal and region of origin in migratory species	Genomic data* or targeted genomic panels: GT-sequencing, Fluidigm assay, Sequenced genome-wide microsatellites from source, and sink populations	Evidence of harvest of protected cod population outside of MPA boundaries (Sinclair-Waters et al., 2018a)
	Parentage and sibship analyses to investigate dispersal and source/sink dynamics	Adequate sampling of adults and juveniles may be a limiting step for GSI and parentage/sibship analyses	Assignment of neon goby to three source populations revealed few long-distance dispersers and low connectivity along the Belize Barrier Reef (D’Aloia et al., 2022)
	Genetics can be integrated with biophysical and habitat models for increased confidence in models		Australasian Snapper (*Chrysophrys auratus*) within Cape Rodney to Okakari Marine Reserve contributes juveniles to the surrounding area based on parentage assignment using 17 microsatellites (Le Port et al., 2017)
Characterization of aquatic and microbial communities; detection of pathogens (e.g., marine bacteria, viruses, and fungi)	Metagenomics and metabarcoding; monitoring sites can be developed to create time series across seasons/years	eDNA/eRNA/metagenomic short- and/or long-read sequences (e.g., Illumina or Nanopore); associated environmental metadata	eDNA monitoring Scorpion State Marine Reserve detected 23 more fish species than visual surveys (Gold et al., 2021)
	An example monitoring plan could sample triplicate one-liter samples at select monitoring sites on a seasonal or annual basis to create a time series of monitoring stations		Using COI and 18S rRNA sequences, Sawaya et al. (2019) assessed eukaryotic diversity across multiple trophic levels in the Florida Keys National Marine Sanctuary. 18S recovered 785 genera while COI sequences only recovered 115 genera, and only 33 genera overlapped between both datasets.
			Bruce et al. (2012) identified a higher abundance of archaeal, viral, and pathogenic bacterial gene sequences in unprotected reefs, while a higher abundance of prokaryotic genes related to photosynthesis were sequenced from water samples from an MPA.
Measure impacts of anthropogenic stressors on species within MPAs	Changes in allele frequency, gene expression, epigenetic markers, or eRNA markers	Genomic data*	Genomic signatures of fishery induced selection (i.e., size selective harvest) could be detected using low-coverage whole-genome resequencing (lcWGS) (Therkildsen et al., 2019)
		Transcriptomics (e.g., RNA-sequencing)	Expression of stress-related genes (*via* RNA-sequencing) were higher in crab outside MPA’s boundaries consistent with higher contaminant levels (Baratti et al., 2022)
		Epigenomics (e.g., DNA methylation *via* whole-genome bisulfite sequencing)	eRNA has the potential to provide novel monitoring approaches, including the ability to assess the health status of organisms and communities (Yates et al., 2021)
		eRNA collected from water samples	
Quantifying changes in effective population size and intraspecific diversity	Changes in effective population size (e.g., software program LinkNe)	LinkNe: SNP based genomic data* (>1,000 loci) and linkage map information with adequate sampling of populations (>40 individuals) (Hollenbeck et al., 2016)	Quantifying temporal trends in contemporary effective population size (*N*_e_) using SNP data in marine species (e.g., Kess et al., 2019; Lehnert et al., 2019)
	Close-kin mark recapture (CKMR)	CKMR: Sequencing based genotyping (genomic data*) or targeted panel (i.e., sequenced microsatellites) capable of assigning parentage. Sampling should include a large number of adults and juveniles (or multiple age classes) over multiple years to identify parent-offspring pairs. Sample size dependent on species (e.g., highly abundant species require large number of samples; see Bravington et al., 2016)	Estimation of absolute abundance and population trends using close-kin mark recapture (CKMR) using sequencing approaches, including in highly mobile marine fish (e.g., Bravington et al., 2016)
Forecast population change and vulnerability under future climate change	Identification of loci associated with the current climate and forecasting genetic change required to match future climate (i.e., genetic offset or genomic vulnerability)	Genomic data* or transcriptomics data (RNA-sequencing), with current and future climate data using predictive models	Using SNP array data, southern populations of Arctic charr were predicted to be most vulnerable to climate change (Layton et al., 2021)
			Using transcriptomic based SNP data, simulations revealed the likely extinction of a coral population under severe climate change scenario (Bay et al., 2017b)

*Genomic data can include SNPs (often thousands to millions), structural variants (e.g., copy number variation), and sequenced microsatellites derived from methods such as low-coverage whole-genome sequencing (lcWGS), pooled sequencing at the population level (PoolSeq), restriction site associated DNA sequencing (RAD-seq), SNP arrays, and other methods.

Multi-species genomic approaches can provide information for understanding interactions between various levels of the ecosystem within an MPA network (Andrello et al., 2022). For example, comparative genomic approaches can help prioritize species (or populations) needing protection by identifying those that are most vulnerable to threats (Zoonomia Consortium 2020) or those with the greatest likelihood of recovery (Beichman et al., 2019). In contrast to the current genomic approaches relying on single reference genomes, a pangenomics approach which focuses not only on sequence diversity but also on the structural diversity of genomes would improve the quantification of adaptive diversity and predictions of future changes (Brockhurst et al., 2019).

The non-invasive nature of eDNA metabarcoding sampling makes it an ideal alternative to direct, in some cases destructive, monitoring techniques, and a strong candidate for real-time autonomous sampling (e.g., Hansen et al., 2020). Similarly, metagenomic sequencing of microorganisms provides a tool for monitoring ecological quality (Fruehe et al., 2021) and ecosystem services within MPAs (Curry and Ausubel 2021). For example, eDNA of benthic microbial community composition coupled with machine learning approaches has shown promise for improving indices of environmental impacts (Fruehe et al., 2021). While metabarcoding is now routinely applied by researchers to answer ecological questions, its uptake by managers and commercial industries is lagging behind (Curry and Ausubel 2021). Yet eDNA has great potential to complement existing biodiversity sampling approaches such as trawling and electrofishing (e.g., Afzali et al., 2021; Gold et al., 2021; He et al., 2022), and eventually, potentially replace these traditional methods in sensitive habitats often associated with protection by MPAs.

To contribute to real-time monitoring for the blue economy, it has even been proposed that ships could be designed to filter water along their transport routes, potentially covering large geographic areas and making the data readily available to resource managers and the public (Curry and Ausubel 2021). The “real-time” inference drawn from eDNA research can be further enhanced using environmental RNA (eRNA), which offers additional power to these eDNA approaches due to the high turnover rate of RNA compared to DNA (Yates et al., 2021). eRNA can enhance spatio-temporal resolution compared to traditional eDNA methods, as it primarily reflects physiologically active organisms that are in close proximity to sampling locations, and it can provide novel monitoring approaches, such as the ability to assess the health status of organisms and communities within a more focal area (Cristescu 2019; Yates et al., 2021).

## Conclusion

Molecular omics technologies offer significant potential to improve and transform marine conservation planning, and recognition of its importance for monitoring and providing real-time information for the blue economy is quickly taking hold. The ability to use millions of genome-wide markers to delineate populations, assess connectivity, and determine environmental drivers is invaluable when designing networks of MPAs and subsequently managing and monitoring them. While the fields of genomics and eDNA are perpetually evolving, this should not be seen as a reason to refrain from their operationalization now. The data and sequences generated can be incorporated into future or expanded studies, be used as the basis for monitoring programs, or provide historical baselines as we enter an era of unprecedented biodiversity change. The adoption of these techniques toward marine conservation will allow targeted planning of protected areas and effective monitoring of changes in populations from anthropogenic and climate impacts, together enabling targeted and adaptive marine conservation.

## Data Availability

The original contributions presented in the study are included in the article/Supplementary Material; further inquiries can be directed to the corresponding author.
